# First record of *Eutrichosiphum sinense* Raychaudhuri (Hemiptera, Aphididae, Greenideinae) from South Korea, with an illustrated key to the Korean Greenideinae species

**DOI:** 10.3897/BDJ.14.e190624

**Published:** 2026-07-10

**Authors:** Yejin Kang, Wonhoon Lee

**Affiliations:** 1 Department of Plant Medicine, Gyeongsang National University, Jinju, Republic of Korea Department of Plant Medicine, Gyeongsang National University Jinju Republic of Korea; 2 Institute of Agriculture & Life Science, Gyeongsang National University, Jinju, Republic of Korea Institute of Agriculture & Life Science, Gyeongsang National University Jinju Republic of Korea

**Keywords:** Greenideinae, *
Eutrichosiphum
*, New record, South Korea

## Abstract

**Background:**

The subfamily Greenideinae (Hemiptera, Aphididae) comprises approximately 180 species in 16 genera worldwide. It is divided into three tribes: Cervaphidini, Greenideini, and Schoutedeniini, with the majority of species assigned to Greenideini. In South Korea, seven species representing four genera have been recorded to date.

**New information:**

In 2025, *Eutrichosiphum sinense* Raychaudhuri, 1956 was collected from *Castanopsis sieboldii* in Samgye-ri, Naedong-myeon, Jinju-si, South Korea (35.1477, 128.0448). This species is reported here for the first time from South Korea. Illustrations of apterous viviparous females, together with morphological descriptions, measurements, host plant information, and distributional data, are provided.

## Introduction

The subfamily Greenideinae (Hemiptera, Aphididae) comprises approximately 180 species in 16 genera worldwide (Favret and Aphid Taxon Community 2025). It is currently divided into three tribes: Cervaphidini van der Goot, 1917; Greenideini Baker, 1920; and Schoutedeniini Nieto Nafría, Mier Durante & Pérez Hidalgo, 2011, with the majority of species assigned to Greenideini (Favret and Aphid Taxon Community 2025).

Members of Greenideinae are morphologically characterized by pointed or bifurcated dorsal setae and elongate, swollen (in apterae) siphunculi bearing very long, hair-like setae. Ecologically, most species feed on woody hosts within Fagales, Sapindales, and Malvales (e.g., Fagaceae, Sabiaceae, Myrtaceae, Tiliaceae, and Betulaceae).

To date, seven species in four genera have been reported from South Korea: one in Cervaphidini, *Cervaphis quercus* Takahashi, 1918; six in Greenideini, *Greenidea kuwanai* (Pergande, 1906); *G. nipponica* Suenaga, 1934; *G. nigra* (Maki, 1917); *G. prunicola* Ghosh, Banerjee & Raychaudhuri, 1971; *Mollitrichosiphum luchuanum* (Takahashi, 1930); *Eutrichosiphum pasaniae* (Okajima, 1908) (Kang and Lee 2025a, Kang and Lee 2025b, Kang and Lee 2025c, Paik 1965, Paik 1972).

During surveys conducted between 2023 and 2025, we collected additional Greenideinae species from several regions of South Korea. These surveys revealed previously unrecorded host plant associations and led to the discovery of one species newly recorded for the Korean fauna, *Eutrichosiphum sinense* Raychaudhuri, 1956. In addition to the new country record, we provide detailed morphological descriptions and illustrations of apterous viviparous females of the Greenideinae species of South Korea, document host plant associations, and present an updated taxonomic key to the eight species of Greenideinae currently known from South Korea.

## Materials and methods

The samples of aphids were collected from *Quercus* spp., *Castanopsis sieboldii*, and *Meliosma myriantha* in four provinces. The leaves and branches of the host plants were mainly observed through the naked eye and collected using an insect aspirator and brush. The collected samples were recorded with collector, location, date and host plant.

The samples were preserved in 95% alcohol and slide glass specimens were mounted on Canada balsam, following the method of Blackmand and Eastop (2000) methods. Images and measurements were taken by using a LEICA (DM3000 LED) or LEICA (CTR6 LED) microscope/camera combination. All voucher specimens were deposited in the Institute of Agriculture & Life Science, Gyeongsang National University.

The following abbreviations are used in morphological features: BL, body length from the head to the end of cauda; Ant., antennae; Ant.I–VI and Ant.VIb, antennal segments I–VI, and basal part of antennal segment VI, respectively; PT, processus terminalis; BDAnt.III, basal diameter of antennal segment III; 2HT, second segment of hind tarsus; HFM, hind femur; HTB, hind tibia; SIPH, siphunculus; SIPH BW, basal width of siphunculi; SIPH MW, maximum width of siphunculi; SIPH DW, distal width of siphunculi; Cauda BW, basal width of cauda; URS, ultimate rostral segment; and Head cephalic bp, Head cephalic branched process.

## Taxon treatments

### Cervaphis quercus

Takahashi, 1918

FA809887-4F28-58FB-A5A4-0EDEC0E373AF

Diverosiphum kunugii Shinji, 1922: 791.
Cervaphis kunugi Shinji, 1941: 698.

#### Materials

- **Type status:** Other material. **Occurrence:** catalogNumber: coll#.20240801-010; recordedBy: Yejin Kang; individualCount: 14; sex: female; lifeStage: apterous female; occurrenceStatus: present; associatedOccurrences: host: Quercus acutissima; occurrenceID: C6A179AF-4562-5F39-96F8-0556ECEA6B8D; **Taxon:** scientificName: *Cervaphis quercus*; class: Insecta; order: Hemiptera; family: Aphididae; genus: Cervaphis; taxonomicStatus: accepted; **Location:** higherGeography: East Asia; country: South Korea; countryCode: KR; stateProvince: Jeju-do; municipality: Seongnam-si; locality: Gumi-dong, Bundang-gu; verbatimLatitude: 37.3460; verbatimLongitude: 127.1201; geodeticDatum: WGS84; georeferenceProtocol: label; **Identification:** identifiedBy: Yejin Kang; dateIdentified: 2024; **Event:** samplingProtocol: hand collected; year: 2024; month: 8; day: 1; **Record Level:** language: en; rightsHolder: Gyeongsang National University; collectionID: GNU; collectionCode: Insects; basisOfRecord: PreservedSpecimen; **Material Entity:** preparations: microscope slide

#### Description

**Figure 1.**
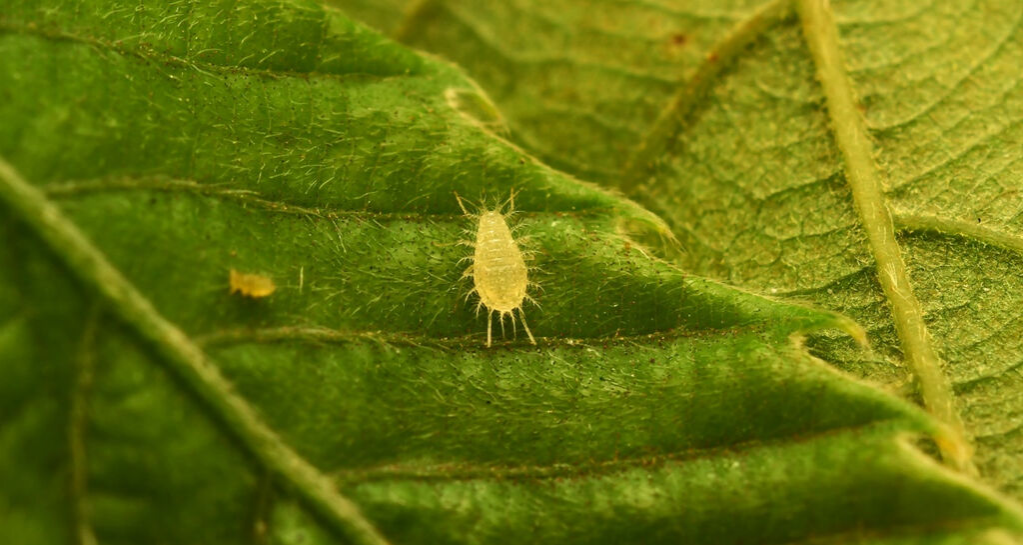
Apterous viviparous female of *Cervaphis quercus* on *Quercus acutissima*.

**Figure 2.**
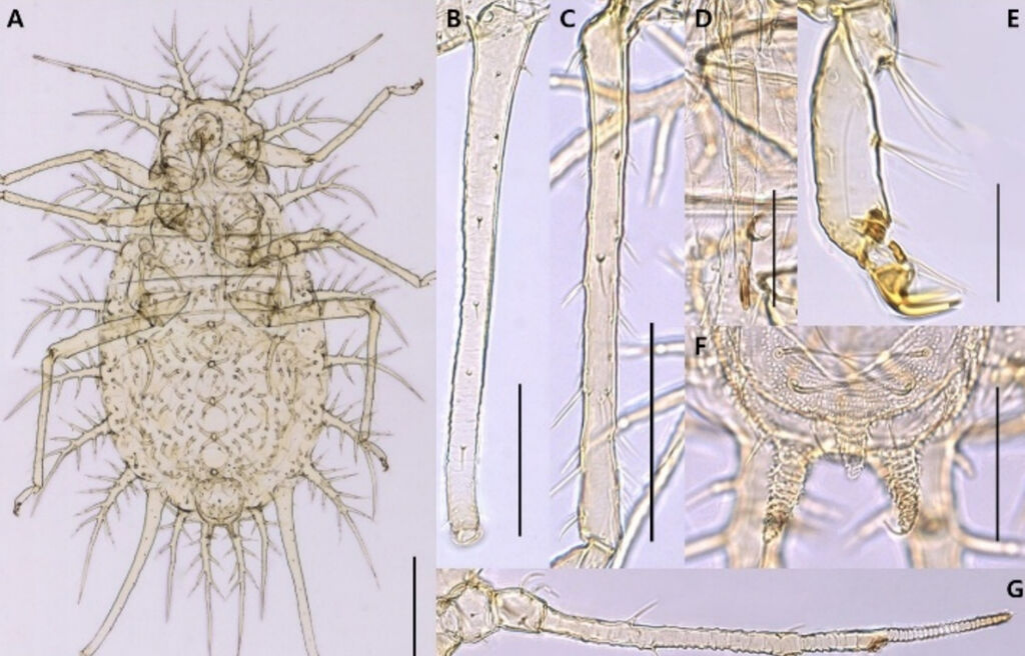
Apterous viviparous female of *Cervaphis quercus*. **A** Whole body; **B** SIPH; **C** HTB; **D** URS; **E** 2HT; **F** Cauda; **G** Whole Antenna. Scale bar: 500 µm (A); 200 µm (B, C); 100 µm (D, F, G); 50 µm (E).

**Apterous viviparous female** (Table 1). Color in life. Body pale yellow, except Ant.Ⅳ; dark brown distal end of Ant.Ⅳb, PT; (Fig. 1). Morphology. Body pale yellow, oblong oval, 1.49-1.76 mm long; with 10 pairs of branched processes in marginal parts of body (Fig. 2A). Head and prothorax fused together with 1 pair of cephalic branched processes, 0.30-0.41 mm long; eye of three facets. Antennae 4-segmented, pale yellow except distal end of PT brown; 0.56-0.65 mm long (Fig. 2G). Ant.Ⅲ with 3-4 setae, imbricated on distal 2/3; Ant.Ⅳb imbricated with 1 seta; PT 0.12-0.15 mm long, intensely imbricated with 2-4 short apical setae, 1.18-1.60 times as long as Ant.Ⅳb; longest seta on Ant.III 0.03-0.04 mm long. URS 0.18-0.22 mm long, with 3-7 accessory setae; URS not passing the hind coxae (Fig. 2D). Hind femur and hind tibia smooth, with long and short setae together (Fig. 2C); 1HT with 3-4 setae; 2HT pale, smooth with 5-7 setae (Fig. 2E). Abdomen smooth ventrally, many non-branched processes in dorsum; ventral setae long and pointed; dorsal setae short and pointed. SIPH pale, bearing 11-16 setae (Fig. 2B); 0.41-0.50 times as long as BL, 7.53-10.20 times as long as basal width of SIPH. Cauda with 6 setae (Fig. 2F); 0.40-0.56 times as long as width.

#### Distribution

South Korea, Japan, China, Taiwan, Thailand, India, Laos. (Miyazaki et al. 2016)

#### Host plants

*Quercus acutissima* (in this study), *Q. aliena*, *Q. kerrii*, *Q. variabilis*, *Castanea crenata*, *C. mollissima*. (Okamoto and Takahashi 1927, Paik 1965)

### Eutrichosiphum pasaniae

(Okajima, 1908)

DC2ED268-6154-5424-BA89-1461DA6F1E06

Trichosiphum pasaniae Okajima, 1908: 23.
Trichosiphum lithocarpae Takahashi, 1921: 68.
Eutrichosiphum lithocarpi Takahashi, 1930: 322.
Eutrichosiphum malayense Takahashi, 1950: 589.
Eutrichosiphum (Eutrichosiphum) vandergooti Raychaudhuri, 1956:11, 19.
Eutrichosiphum passaniae Chakrabarti, Ghosh & Raychaudhuri, 1972:390.

#### Materials

- **Type status:** Other material. **Occurrence:** catalogNumber: coll#.20230829-010; recordedBy: Yejin Kang; individualCount: 8; sex: female; lifeStage: apterous female; occurrenceStatus: present; associatedOccurrences: host: Castanopsis sieboldii; occurrenceID: 87511002-01D2-5A6D-9D5B-E115ED6BFD2B; **Taxon:** scientificName: *Eutrichosiphum pasaniae*; class: Insecta; order: Hemiptera; family: Aphididae; genus: Eutrichosiphum; taxonomicStatus: accepted; **Location:** higherGeography: East Asia; country: South Korea; countryCode: KR; stateProvince: Jeju-do; municipality: Seogwipo-si; locality: Gamsan-ri, Andeok-myeon; verbatimLatitude: 33.2484; verbatimLongitude: 126.3522; geodeticDatum: WGS84; georeferenceProtocol: label; **Identification:** identifiedBy: Yejin Kang; dateIdentified: 2023; **Event:** samplingProtocol: hand collected; year: 2023; month: 8; day: 29; **Record Level:** language: en; rightsHolder: Gyeongsang National University; collectionID: GNU; collectionCode: Insects; basisOfRecord: PreservedSpecimen; **Material Entity:** preparations: microscope slide
- **Type status:** Other material. **Occurrence:** catalogNumber: coll#.20240618-010; recordedBy: Yejin Kang; individualCount: 6; sex: female; lifeStage: apterous female; occurrenceStatus: present; associatedOccurrences: host: Castanopsis sieboldii; occurrenceID: B06D3607-3C10-5083-9857-1F2B0A8B4223; **Taxon:** scientificName: *Eutrichosiphum pasaniae*; kingdom: Animal; phylum: Arthropoda; class: Insecta; order: Hemiptera; family: Aphididae; genus: Eutrichosiphum; taxonomicStatus: accepted; **Location:** higherGeography: East Asia; country: South Korea; countryCode: KR; stateProvince: Jeju-do; municipality: Seogwipo-si; locality: Gamsan-ri, Andeok-myeon; verbatimLatitude: 33.2484; verbatimLongitude: 126.3522; geodeticDatum: WGS84; georeferenceProtocol: label; **Identification:** identifiedBy: Yejin Kang; dateIdentified: 2024; **Event:** samplingProtocol: hand collected; year: 2024; month: 6; day: 18; **Record Level:** language: en; rightsHolder: Gyeongsang National University; collectionID: GNU; collectionCode: Insects; basisOfRecord: PreservedSpecimen

#### Description

**Figure 3.**
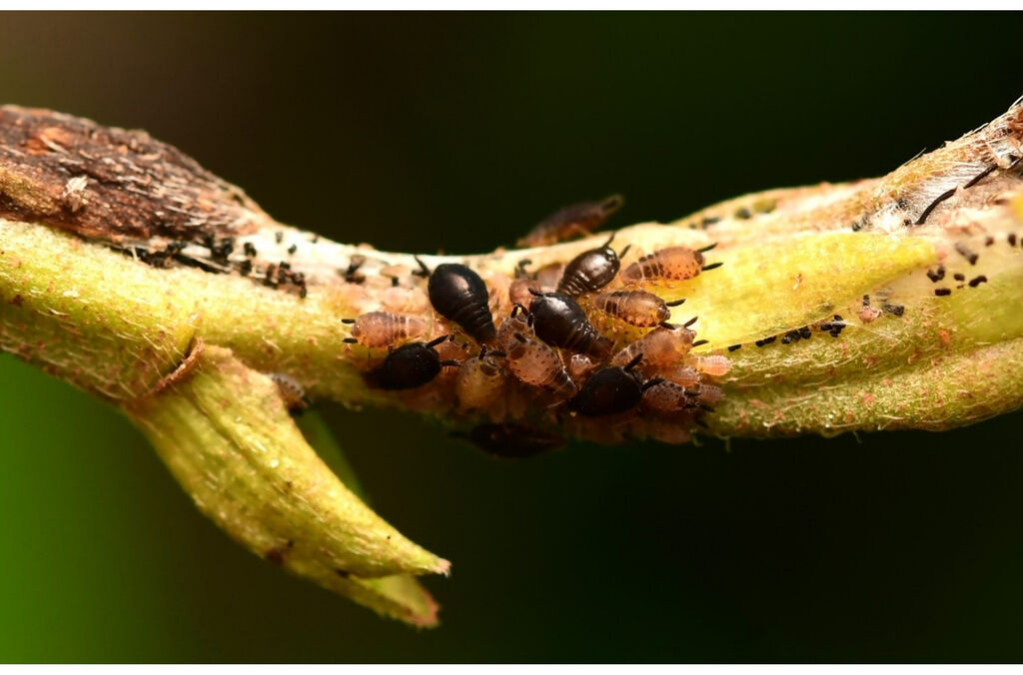
Apterous viviparous female of *Eutrichosiphum pasaniae* on *Castanopsis sieboldii*.

**Figure 4.**
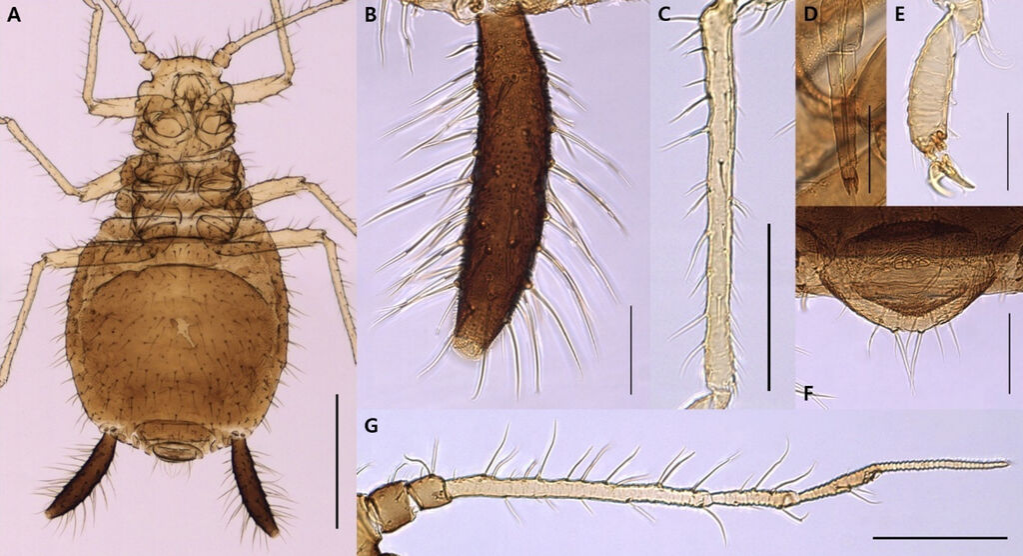
Apterous viviparous female of *Eutrichosiphum pasaniae*. **A** Whole body; **B** SIPH; **C** HTB; **D** URS; **E** 2HT; **F** Cauda; **G** Whole Antenna. Scale bar: 500 µm (A); 200 µm (C, G); 100 µm (B, D, F); 50 µm (E).

**Apterous viviparous female** (Table 2). Color in life. Body semi-glossy dark brown, except legs, anterior head, antennae; dark brown distal half of Ant.Ⅵ and Ant.Ⅴ (Fig. 3). Morphology. Body brown pear-shaped, 1.45-1.64 mm long (Fig. 4A). Head and prothorax fused together. Antennae 5-segmented, pale brown except Ant.Ⅰ-Ⅱ; distal half of Ant.Ⅳ, Ⅴb brown; 0.69-1.00 mm long (Fig. 4G). Ant.Ⅲ with 12-23 setae, weakly imbricated on distal 2/3; Ant.Ⅳ imbricated with 3-6 setae; Ant.Ⅴ intensely imbricated with 3-5 setae on Ant.Ⅴb; PT 0.14-0.23 mm long, with 1-4 short apical setae, 1.40-1.97 times as long as Ant.Ⅴb. Ant.Ⅲ 0.78-1.05 times as long as head width across eyes; longest seta on Ant.III 0.09-0.11 mm long. URS 0.14-0.19 mm long, with 7-10 accessory setae; URS passing hind coxae (Fig. 4D). Hind femur smooth, except distal 1/3 spinulose; hind tibia smooth except imbricated on distal 1/4 (Fig. 7C); 2HT imbricated with 6-8 setae (Fig. 4E). Abdomen spinulose, long and pointed setae dorsally; marginal area of abdomen spinulose, with long and short setae ventrally. SIPH dark brown, with many long setae and spinulose without reticulation at the base (Fig. 4B); 0.19-0.34 times as long as BL, 5.39-9.90 times as long as basal width of SIPH. Cauda with 6-8 (mostly 7) setae (Fig. 4F); 0.29-0.40 times as long as width of cauda.

#### Distribution

South Korea, Japan, Taiwan, China, India, Vietnam, Nepal (Paik 1972, Miyazaki et al. 2016).

#### Host plants

*Castanopsis sieboldii* (in this study), *C. carlesii*, *C. fabri*, *C. tribuloides*, *C. uraiana*, *Clerodendrum cyrtophyllum*, *Ilex ficoidea*, *Lithocarpus* sp., *Quercus griffithii*, *Q. acuta*, *Q. serrata*. (*Okajima 1908, Li et al. 2020*)

### Eutrichosiphum sinense

Raychaudhuri, 1956

8DF37D6C-60DB-5014-B20D-C9EF15095280

Eutrichosiphum (Eutrichosiphum) sinense Raychaudhuri, 1956: 10, 18.
Eutrichosiphum sinense Raychaudhuri, 1956 in Eastop & Hille Ris Lambers, 1976: 198.

#### Materials

- **Type status:** Other material. **Occurrence:** catalogNumber: coll#.20250524-001; recordedBy: Yejin Kang; individualCount: 5; sex: female; lifeStage: apterous female; occurrenceStatus: present; associatedOccurrences: host: Castanopsis sieboldii; occurrenceID: 63204579-F101-5EC6-A307-D797F9294351; **Taxon:** scientificName: *Eutrichosiphum sinense*; class: Insecta; order: Hemiptera; family: Aphididae; genus: Eutrichosiphum; taxonomicStatus: accepted; **Location:** higherGeography: East Asia; country: South Korea; countryCode: KR; municipality: Jinju-si; locality: Samgye-ri, Naedong-myeon; verbatimLatitude: 35.1477; verbatimLongitude: 128.0448; geodeticDatum: WGS84; georeferenceProtocol: label; **Identification:** identifiedBy: Yejin Kang; dateIdentified: 2025; **Event:** samplingProtocol: hand collected; year: 2025; month: 5; day: 24; **Record Level:** language: en; rightsHolder: Gyeongsang National University; collectionID: GNU; collectionCode: Insects; basisOfRecord: PreservedSpecimen; **Material Entity:** preparations: microscope slide

#### Description

**Figure 5.**
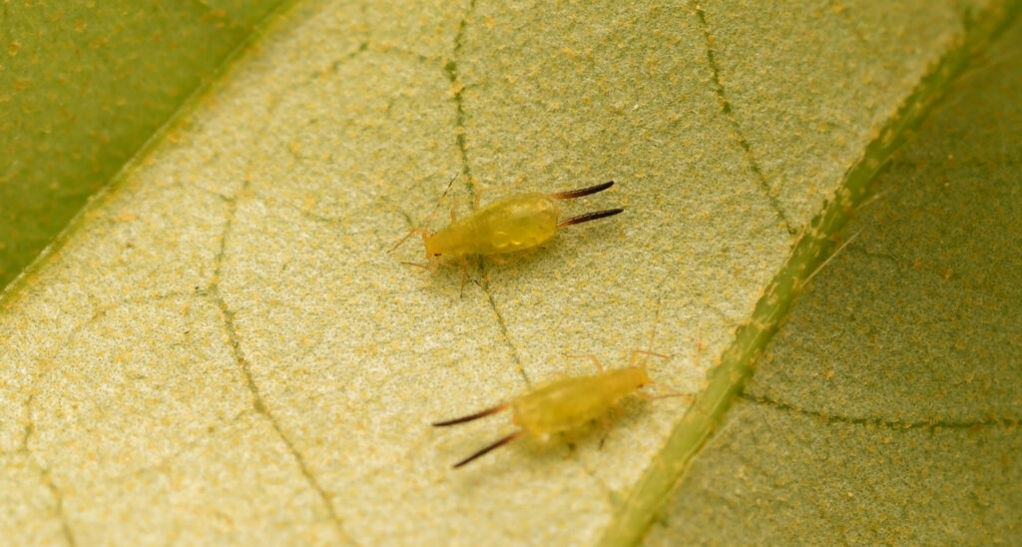
Apterous viviparous female of *Eutrichosiphum sinense* on *Castanopsis sieboldii*.

**Figure 6.**
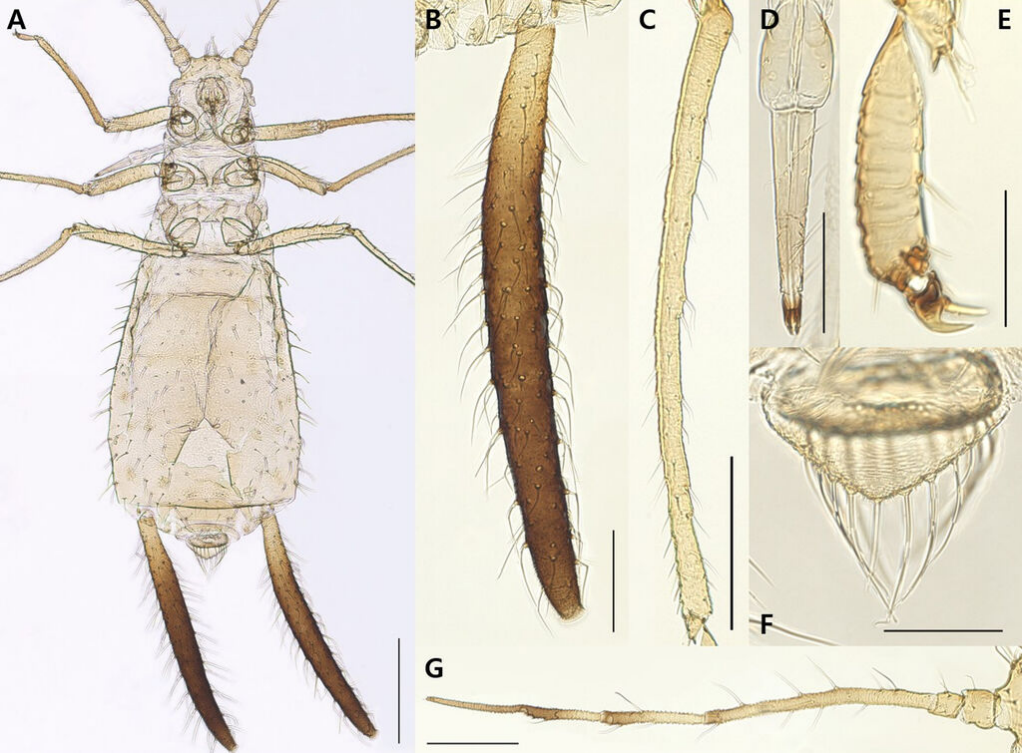
Apterous viviparous female of *Eutrichosiphum sinense*. **A** Whole body; **B** SIPH; **C** HTB; **D** URS; **E** 2HT; **F** Cauda; **G** Whole Antenna. Scale bar: 500 µm (A); 200 µm (B, C, G); 100 µm (D, F); 50 µm (E).

**Apterous viviparous female** (Table 3). Color in life. Body pale yellowish green, antennae; dark brown the end of Ant.Ⅲ, distal half of Ant. Ⅳ, Ant.Ⅴb and PT. SIPH dark brown, except basal parts (Fig. 5). Morphology. Body pale brown elongated, 2.22-2.51 mm long (Fig. 6A). Head and prothorax fused together. Antennae 5-segmented, pale brown except distal end of Ant.Ⅲ, the half of Ant.Ⅳ, Ⅴb and PT brown; 1.21-1.40 mm long (Fig. 6G). Ant.Ⅲ with 15-19 setae, imbricated in general; Ant.Ⅳ and Ant.Ⅴ intensely imbricated with 4-5 setae and 3 setae on Ant.Ⅴb; PT 0.21-0.25 mm long, with 3-4 short apical setae, 1.25-1.55 times as long as Ant.Ⅴb. Ant.Ⅲ 1.23-1.34 times as long as head width across eyes; longest seta on Ant.III 0.09-0.11 mm long. URS 0.17-0.19 mm long, with 8-12 accessory setae; URS not reaching the hind coxae (Fig. 6D). Hind coxa spinulose; hind femur spinulose in general, and reticulated distally; hind tibia smooth except spinulose on distal 1/3 (Fig. 6C); 2HT imbricated with 4-9 setae (Fig. 6E). Abdomen spinulose, with partially elliptical reticulations (also in thorax), pointed and bifurcated setae dorsally; spinulose with long and short setae ventrally. SIPH dark brown except the basal part, with many long setae and spinulose without reticulation at the base (Fig. 6B); 0.45-0.53 times as long as BL, 15.10-19.08 times as long as basal width of SIPH. Cauda with 7-10 (mostly 9) setae (Fig. 6F); 0.42-0.51 times as long as width of cauda.

#### Distribution

South Korea (new record), Japan, China, India, Java (Raychaudhuri 1956, Singh and Singh 2017, Noordam 1994, Miyazaki et al. 2016).

#### Host plants

*Castanopsis sieboldii* (in this study), *C. eyrei*, *C. ferox*, *C. javanica*, *C. mollissima*, *Lithocarpus ovalis*, *Pterospermum* sp. (Noordam 1994, Singh and Singh 2017)

### Greenidea kuwanai

(Pergande, 1906)

7476E113-EB76-5F4F-9A5D-40DA35A95D1F

Trichosiphum kuwanae Pergande, 1906: 209.
Trichosiphum kuwanea Okajima, 1908: 20.

#### Materials

- **Type status:** Other material. **Occurrence:** catalogNumber: coll#.20240607-001; recordedBy: Yejin Kang; individualCount: 11; sex: female; lifeStage: apterous female; occurrenceStatus: present; associatedOccurrences: host: Quercus variabilis; occurrenceID: 6B2B7D6D-1B52-5667-B934-6B2446DC53C5; **Taxon:** scientificName: *Greenidea kuwanai*; class: Insecta; order: Hemiptera; family: Aphididae; genus: Greenidea; subgenus: Trichosiphum; taxonomicStatus: accepted; **Location:** higherGeography: East Asia; country: South Korea; countryCode: KR; municipality: Jinju-si; locality: Gajwa-dong; verbatimLatitude: 35.1549; verbatimLongitude: 128.1005; geodeticDatum: WGS84; georeferenceProtocol: label; **Identification:** identifiedBy: Yejin Kang; dateIdentified: 2024; **Event:** samplingProtocol: hand collected; year: 2024; month: 6; day: 7; **Record Level:** language: en; rightsHolder: Gyeongsang National University; collectionID: GNU; collectionCode: Insects; basisOfRecord: PreservedSpecimen; **Material Entity:** preparations: microscope slide

#### Description

**Figure 7.**
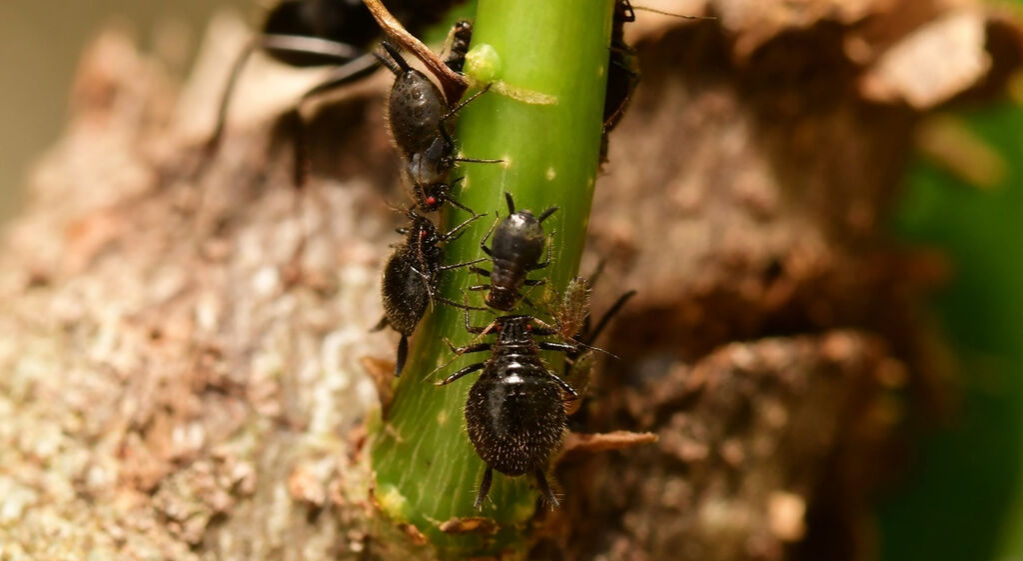
Apterous viviparous female of *Greenidea kuwanai* on *Quercus myrsinifolia*.

**Figure 8.**
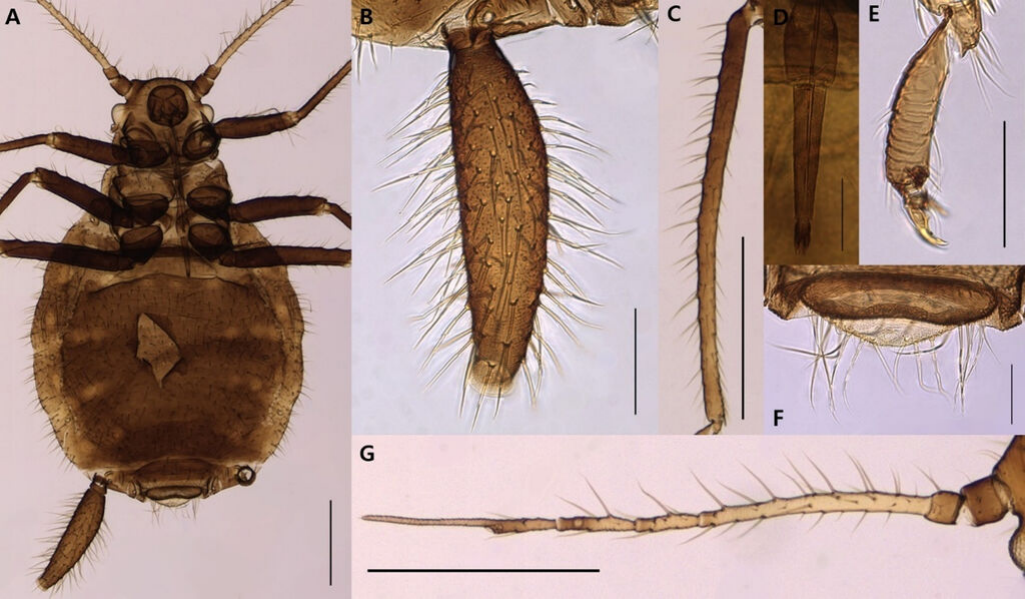
Apterous viviparous female of *Greenidea kuwanai*. **A**, Whole body; **B** SIPH; **C** HTB; **D** URS; **E** 2HT; **F** Cauda; **G** Whole Antenna. Scale bar: 500 µm (A, C, G); 200 µm (B); 100 µm (D, F, E).

**Apterous viviparous female** (Table 4). Color in life. Body glossy black or dark brown, except antennae; dark brown Ant.Ⅰ, Ⅱ; distal end of Ant.Ⅲ, Ⅳ; distal half of Ant.Ⅴ, entire segment Ⅵ; eyes red in life (Fig. 7). Morphology. Body dark brown pear-shaped, 2.10-2.75 mm long (Fig. 8A). Head and prothorax fused together. Antennae 6-segmented, pale brown except Ant.Ⅰ-Ⅱ, distal ends of Ant.Ⅲ-Ⅴ, PT and distal half of Ant.Ⅵb brown; 1.29-1.81 mm long (Fig. 8G). Ant.Ⅲ with 31-50 setae, weakly imbricated on distal half of Ant.Ⅲ; Ant.Ⅳ-Ⅴ imbricated with 7-13, 4-15 setae; Ant.Ⅵ intensely imbricated with 4-8 setae on Ant.Ⅵb; PT 0.27-0.36 mm long, with 3-4 short apical setae, 1.79-2.29 times as long as Ant.Ⅵb. Ant.Ⅲ 0.77-1.05 times as long as head width across eyes; longest seta on Ant.III 0.12-0.16 mm long. URS 0.23-0.29 mm long, with 13-31 accessory setae; the end of rostral segment completely passing the hind coxae (Fig. 8D). Hind femur smooth, bearing vertical stripes and long and short setae; hind tibia smooth, with many long and short setae together; brighter towards distal part (Fig. 8C); 2HT brown (the same color as the bright part of hind tibia), imbricated with 9-12 setae (Fig. 8E). Abdomen spinulose, with long and short setae together ventrally; dorsal setae long and pointed; SIPH brown (brighter towards distal part), with many long setae, spinulose; reticulated at the base (Fig. 8B); 0.19-0.29 times as long as BL, 5.87-8.36 times as long as basal width of SIPH. Cauda with 6-9 (mostly 8) setae (Fig. 8F); 0.28-0.42 times as long as width of cauda.

#### Distribution

South Korea, Japan, Taiwan, China, Siberia (Okamoto and Takahashi 1927, Miyazaki et al. 2016).

#### Host plants

*Quercus variabilis*, *Q. acutissima*, *Q. acuta*, *Q. aliena*, *Q. myrsinifolia*, *Q. serrata*, *Q. glauca*, *Q. dentata*, *Q. fabri*, *Q. mongolica*, *Castanea mollissima*, *Prunus pseudocerasus* (Paik 1972).

### Greenidea nipponica

Suenaga, 1934

C3D432BD-4494-5A5C-A266-8AB5FFE82D3D

#### Materials

- **Type status:** Other material. **Occurrence:** catalogNumber: coll#.20090805-001; recordedBy: H.-R. Choi; individualCount: 1; sex: female; lifeStage: apterous female; occurrenceStatus: present; associatedOccurrences: host: Quercus aliena; occurrenceID: 77B31ED1-62AB-5198-8271-25785BCBAB4B; **Taxon:** scientificName: *Greenidea nipponica*; kingdom: Animal; phylum: Arthropoda; class: Insecta; order: Hemiptera; family: Aphididae; genus: Greenidea; subgenus: Trichosiphum; taxonomicStatus: accepted; **Location:** higherGeography: East Asia; country: South Korea; countryCode: KR; municipality: Boryeong-si; locality: Wonsando; verbatimLatitude: 36.2759; verbatimLongitude: 126.4259; geodeticDatum: WGS84; georeferenceProtocol: label; **Identification:** identifiedBy: H.-R. Choi; dateIdentified: 2009; **Event:** samplingProtocol: hand collected; year: 2009; month: 8; day: 5; **Record Level:** language: en; rightsHolder: Seoul National University; collectionID: SNU; collectionCode: Insects; basisOfRecord: PreservedSpecimen; **Material Entity:** preparations: microscope slide

#### Description

**Figure 9.**
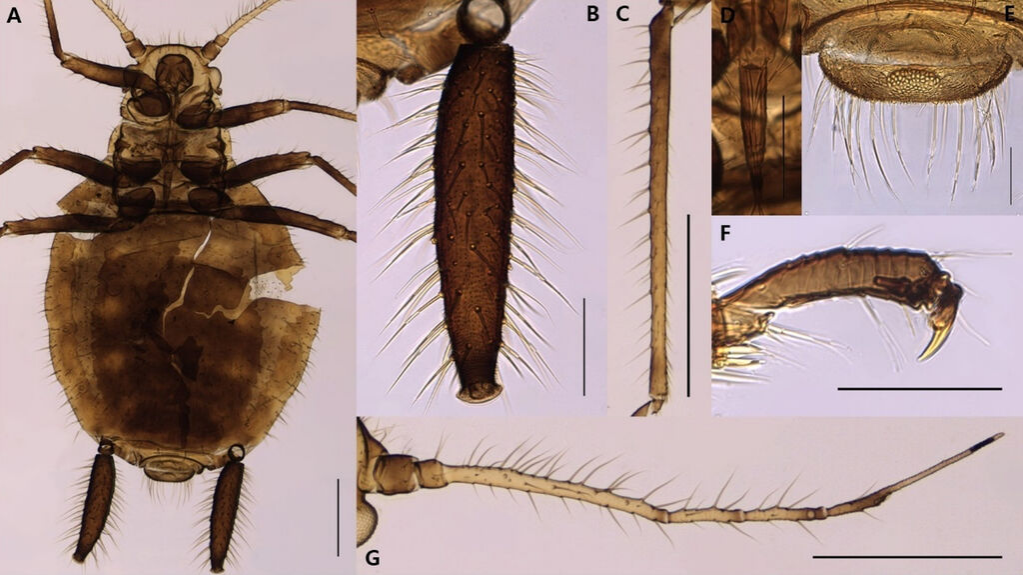
Apterous viviparous female of *Greenidea nipponica*. **A** Whole body; **B** SIPH; **C** HTB; **D** URS; **E** Cauda; **F** 2HT; **G**,Whole Antenna. Scale bar: 500 µm (A, C, G); 200 µm (B, D); 100 µm (F, E).

**Apterous viviparous female** (Table 5). Morphology. Body dark brown pear-shaped, 2.82 mm long (Fig. 9A). Head and prothorax fused together. Antennae 6-segmented, pale brown except Ant.Ⅰ-Ⅱ, distal end of Ant.Ⅲ-Ⅴ and distal half of Ant.Ⅵb brown; 1.71 mm long (Fig. 9G). Ant.Ⅲ with 39 setae, weakly imbricated on distal half of Ant.Ⅲ; Ant.Ⅳ-Ⅴ imbricated with 9, 8 setae; Ant.Ⅵ intensely imbricated with 7 setae on Ant.Ⅵb; PT 0.36 mm long, with 4 short apical setae, 2.28 times as long as Ant.Ⅵb. Ant.Ⅲ 0.95 times as long as head width across eyes; longest seta on Ant.III 0.16 mm long. URS 0.27 mm long, with 18 accessory setae; the end of rostral segment reaching the middle of hind coxae (Fig. 9D). Hind femur smooth, bearing vertical stripes on the underside and long and short setae; hind tibia smooth, with many long and short setae together; brighter towards distal part (Fig. 9C); 2HT brown (darker than the bright part of hind tibia), imbricated with 10 setae (Fig. 9E). Abdomen spinulose, with long and short setae together ventrally; dorsal setae long and pointed; SIPH dark brown (darker basal and distal part), with many long setae and spinulose; reticulated at the base (Fig. 9B); 0.28 times as long as BL, 8.68 times as long as basal width of SIPH. Cauda with 7 setae (Fig. 9F); 0.38 times as long as width of cauda.

#### Distribution

South Korea, Japan, China, Pakistan (Paik 1972, Miyazaki et al. 2016, Hassan et al. 2024).

#### Host plants

*Quercus aliena*, *Q. baloot*, *Q. dentata*, *Q. floribunda*, *Q. leucotrichophora*, *Q. mongolica*, *Q. serrata*, *Q. variabilis* (Hassan et al. 2024, Paik 1972).

### Greenidea nigra

(Maki, 1917)

115EE080-3BCB-5B95-AB69-C839E9ED685E

Trichosiphum nigrum Maki, 1917: 9, 10

#### Materials

- **Type status:** Other material. **Occurrence:** catalogNumber: coll#.20090805-001; recordedBy: H.-R. Choi; individualCount: 12; sex: female; lifeStage: apterous female; occurrenceStatus: present; associatedOccurrences: host: Quercus aliena; occurrenceID: 06054FF3-4699-5C84-891E-85D553CBCCAC; **Taxon:** scientificName: *Greenidea nipponica*; higherClassification: East Asia; kingdom: Animal; phylum: Arthropoda; class: Insecta; order: Hemiptera; family: Aphididae; genus: Greenidea; subgenus: Trichosiphum; taxonomicStatus: accepted; **Location:** country: South Korea; countryCode: KR; municipality: Boryeong-si; locality: Wonsando; verbatimLatitude: 36.2759; verbatimLongitude: 126.4259; geodeticDatum: WGS84; georeferenceProtocol: label; **Identification:** identifiedBy: H.-R. Choi; dateIdentified: 2009; **Event:** samplingProtocol: hand collected; year: 2009; month: 8; day: 5; **Record Level:** language: en; rightsHolder: Seoul National University; collectionID: SNU; collectionCode: Insects; basisOfRecord: PreservedSpecimen; **Material Entity:** preparations: microscope slide

#### Description

**Figure 10.**
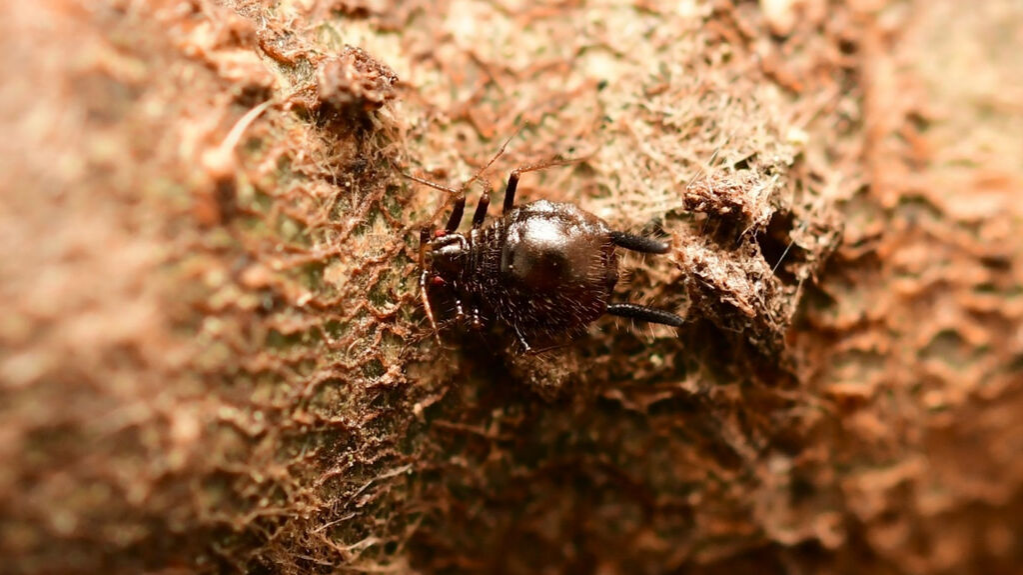
Apterous viviparous female of *Greenidea nigra* on *Quercus glauca*.

**Figure 11.**
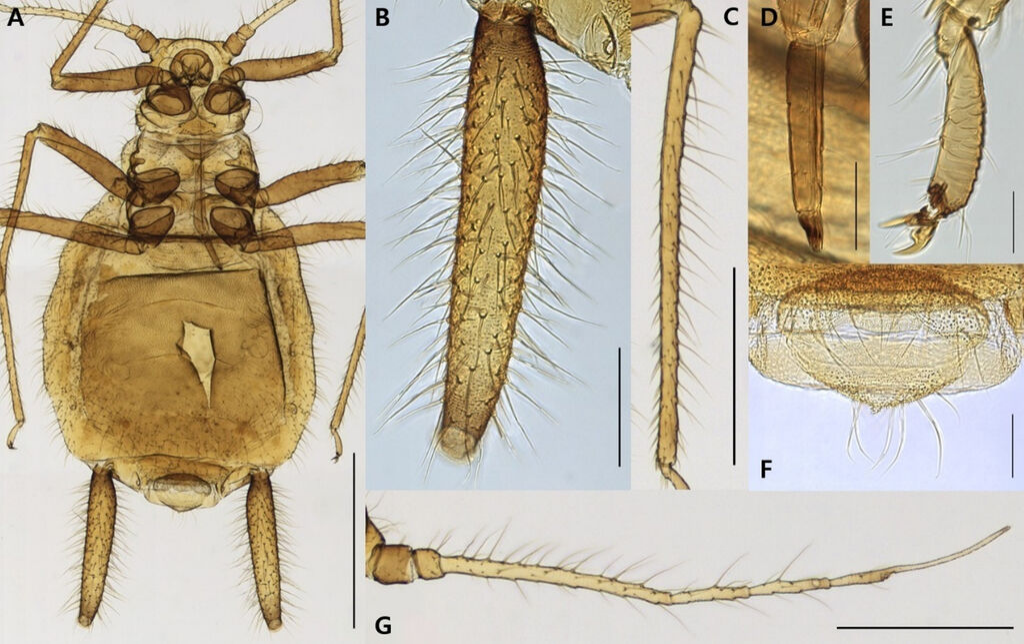
Apterous viviparous female of *Greenidea nigra*. **A** Whole body; **B** SIPH; **C** HTB; **D** URS; **E** 2HT; **F** Cauda; **G** Whole Antenna. Scale bar: 1 mm (A), 500 µm (C, G); 200 µm (B); 100 µm (D, F); 50 µm (E).

**Apterous viviparous female** (Table 6). Color in life. Body glossy reddish dark brown, except tibia and antennae; dark brown Ant.Ⅰ, Ⅱ; distal end of Ant.Ⅲ, Ⅳ; distal half of Ant.Ⅴ, Ⅵb and the basal half of PT; SIPH black in life (Fig. 10). Morphology. Body brown, pear-shaped, 2.11-2.89 mm long (Fig. 11A). Head and prothorax fused together. Antennae 6-segmented, pale brown except Ant.Ⅰ-Ⅱ, distal end of Ant.Ⅴ, Ⅵb brown; 1.52-1.96 mm long (Fig. 11G). Ant.Ⅲ with 35-48 setae, weakly imbricated on distal 1/3 of Ant.Ⅲ; Ant.Ⅳ-Ⅴ imbricated with 6-13, 5-7 setae; Ant.Ⅵ intensely imbricated with 4-7 setae on Ant.Ⅵb; PT 0.28-0.42 mm long, with 3-4 short apical setae, 1.77-2.69 times as long as Ant.Ⅵb. Ant.Ⅲ 0.98-1.08 times as long as head width across eyes; longest seta on Ant.III 0.17-0.21 mm long. URS 0.23-0.26 mm long, with 14-20 accessory setae; basal of 4th rostrum segment passing the middle of hind coxae (Fig. 11D). Hind femur and hind tibia smooth, with many long and short setae together (Fig. 11C); 2HT brown, imbricated with 9-11 setae (Fig. 11E). Abdomen strongly spinulose ventrally; dorsal setae long and pointed; ventral setae short and pointed. SIPH brown, with many long setae and spinulose, reticulated at the base (Fig. 11B); 0.28-039 times as long as BL, 7.54-9.56 times as long as basal width of SIPH. Cauda with 6-8 (mostly 6) setae (Fig. 11F); 0.35-0.44 times as long as width of cauda.

#### Distribution

South Korea, Japan, Taiwan (Kang and Lee 2025b, Miyazaki et al. 2016).

#### Host plants

*Quercus glauca*, *Q. acuta*, *Q. myrsinaefolia*, *Q. salicina*, *Lithocarpus edulis*, *L. formosanus* (Sugimoto 2008, Kang and Lee 2025b, Raychaudhuri 1956).

### Greenidea prunicola

Ghosh, Banerjee & Raychaudhuri, 1971

77B01EFA-28AE-5BC7-AB60-1B3C7FE1FE9E

#### Materials

- **Type status:** Other material. **Occurrence:** catalogNumber: coll#.20240510-010; recordedBy: Yejin Kang; individualCount: 10; sex: female; lifeStage: apterous female; occurrenceStatus: present; associatedOccurrences: host: Castanopsis sieboldii; occurrenceID: BA1CAAA6-9F0C-5485-8165-FEAB4267F524; **Taxon:** scientificName: *Greenidea prunicola*; class: Insecta; order: Hemiptera; family: Aphididae; genus: Greenidea; subgenus: Trichosiphum; taxonomicStatus: accepted; **Location:** higherGeography: East Asia; country: South Korea; countryCode: KR; municipality: Tongyeong-si; locality: Dongho-dong; verbatimLatitude: 34.8417; verbatimLongitude: 128.4301; geodeticDatum: WGS84; georeferenceProtocol: label; **Identification:** identifiedBy: Yejin Kang; dateIdentified: 2024; **Event:** samplingProtocol: hand collected; year: 2024; month: 5; day: 10; **Record Level:** language: en; rightsHolder: Gyeongsang National University; collectionID: GNU; collectionCode: Insects; basisOfRecord: PreservedSpecimen; **Material Entity:** preparations: microscope slide

#### Description

**Figure 12.**
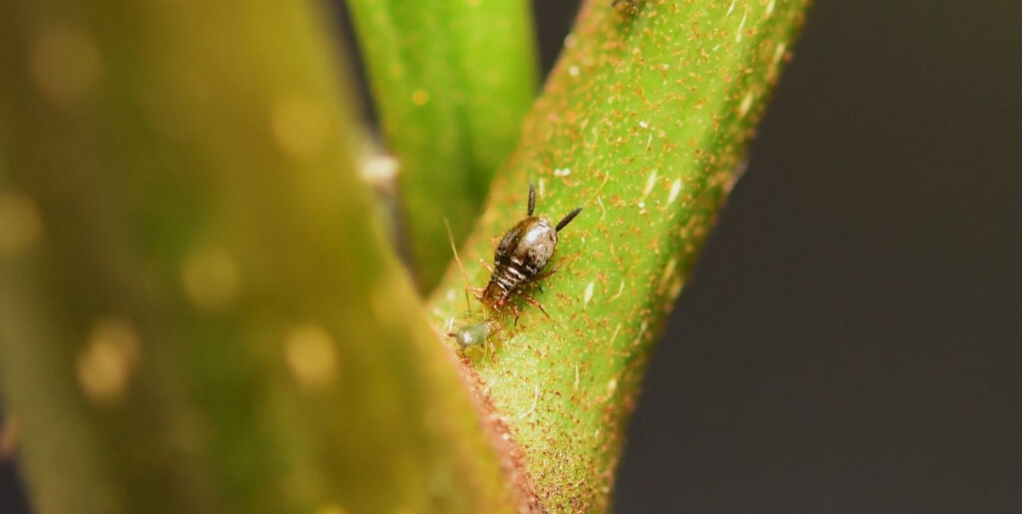
Apterous viviparous female of *Greenidea prunicola* on *Castanopsis sieboldii*.

**Figure 13.**
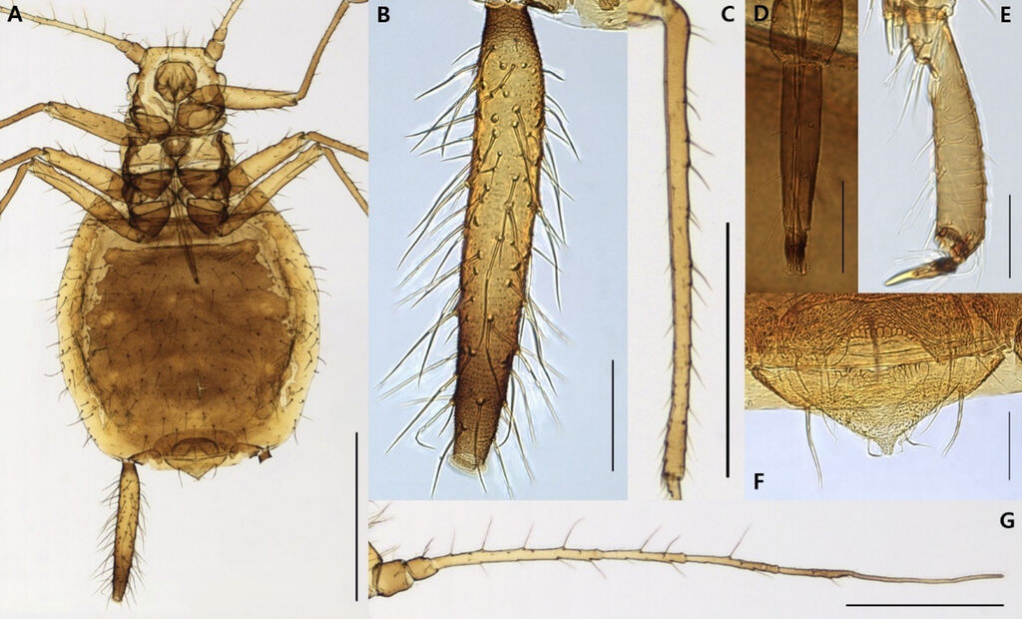
Apterous viviparous female of *Greenidea prunicola*. **A** Whole body; **B** SIPH; **C** HTB; **D** URS; **E** 2HT; **F** Cauda; **G** Whole Antenna. Scale bar: 1 mm (A), 500 µm (C, G); 200 µm (B); 100 µm (D, F); 50 µm (E).

**Apterous viviparous female** (Table 7). Color in life. Body glossy reddish brown; head, legs and antennae paler than abdomen, except distal half of Ⅴ, Ⅵb and the whole PT; SIPH mostly black, reddish dark brown in life (Fig. 12). Morphology. Body brown, pear-shaped, 1.70-2.60 mm long (Fig. 13A). Head fused with prothorax. Antennae 6-segmented, pale brown except Ant.Ⅰ-Ⅱ, distal half of Ⅴ, Ⅵb, and PT brown; 1.47-2.25 mm long (Fig. 13G). Ant.Ⅲ weakly imbricated with 15-21 setae; Ant.Ⅳ-Ⅴ imbricated with 6-9, 5-8 setae; Ant.Ⅵ intensely imbricated with 4-8 setae on Ant.Ⅵb; PT 0.37-0.56 mm long, with 3-4 short apical setae, 2.10-2.51 times as long as Ant.Ⅵb. Ant.Ⅲ 0.80-0.99 times as long as head width across eyes; longest seta on Ant.III 0.10-0.13 mm long. Distal of rostrum passing the hind coxae; URS 0.18-0.23 mm long, with 12-16 accessory setae (Fig. 13D). Hind coxae imbricated, bearing compact spinules on the margins of imbrications; hind femur imbricated; hind tibia transverse imbricated (Fig. 13C); 2HT brown, imbricated with 9-10 setae (Fig. 13E). Abdomen intensely spinulose ventrally; dorsal setae long, mainly bifurcated; ventral setae short and pointed. SIPH brown except the basal and distal part, bearing many long setae and spinulose, reticulated at the base (Fig. 13B); 0.32-0.37 times as long as BL, 9.50-12.03 times as long as basal width of SIPH. Cauda with 6-8 (mostly 8) setae (Fig. 13F); 0.34-0.48 times as long as width of cauda.

#### Distribution

South Korea, China, India (Ghosh et al. 1971, Liu et al. 2013).

#### Host plants

*Castanopsis sieboldii*, *Prunus* spp. (Ghosh et al. 1971)

### Mollitrichosiphum luchuanum

(Takahashi, 1930)

9E4A0046-664B-5135-BACC-65CED546781C

Greenidea luchuana Takahashi, 1930: 322
Mollitrichosiphum (Metatrichosiphon) luchuana Ghosh, 1974: 169
Mollitrichosiphum (Metatrichosiphon) luchuanum Remaudière, G. & M. Remaudière, 1997: 177

#### Materials

- **Type status:** Other material. **Occurrence:** catalogNumber: coll#.20240619-001; recordedBy: Yejin Kang; individualCount: 12; sex: female; lifeStage: apterous female; occurrenceStatus: present; associatedOccurrences: host: Meliosma myriantha; occurrenceID: 33BF5494-77C2-590D-90BE-1D179C1A0252; **Taxon:** scientificName: *Mollitrichosiphum luchuanum*; class: Insecta; order: Hemiptera; family: Aphididae; genus: Mollitrichosiphum; subgenus: Metatrichosiphon; taxonomicStatus: accepted; **Location:** higherGeography: East Asia; country: South Korea; countryCode: KR; municipality: Seogwipo-si; locality: Seogwipo Natural Recreational Forest; verbatimLatitude: 33.3122; verbatimLongitude: 126.4592; geodeticDatum: WGS84; georeferenceProtocol: label; **Identification:** identifiedBy: Yejin Kang; dateIdentified: 2024; **Event:** samplingProtocol: hand collected; year: 2024; month: 6; day: 19; **Record Level:** language: en; rightsHolder: Gyeongsang National University; collectionID: GNU; collectionCode: Insects; basisOfRecord: PreservedSpecimen; **Material Entity:** preparations: microscope slide

#### Description

**Figure 14.**
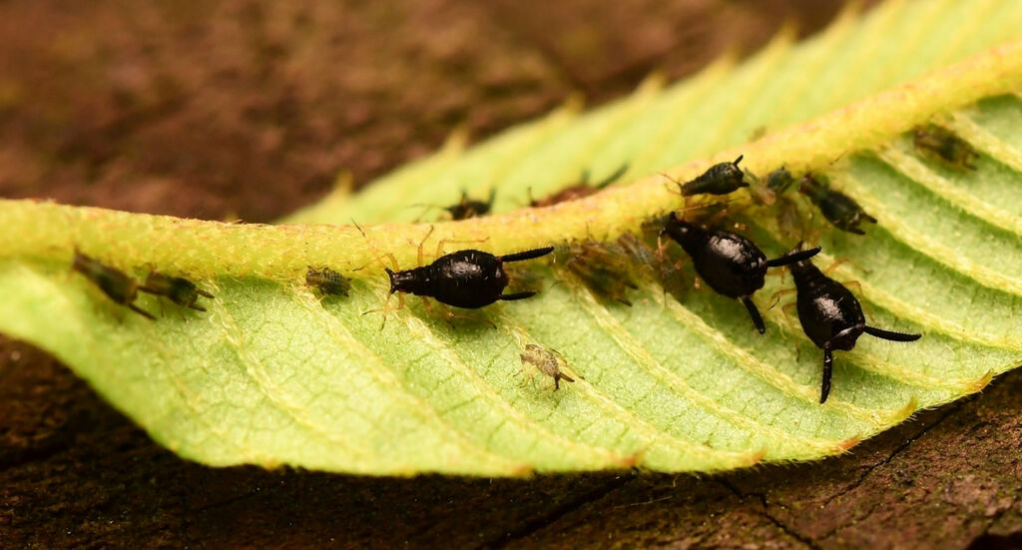
Apterous viviparous female of *Mollitrichosiphum luchuanum* on *Meliosma myriantha*.

**Figure 15.**
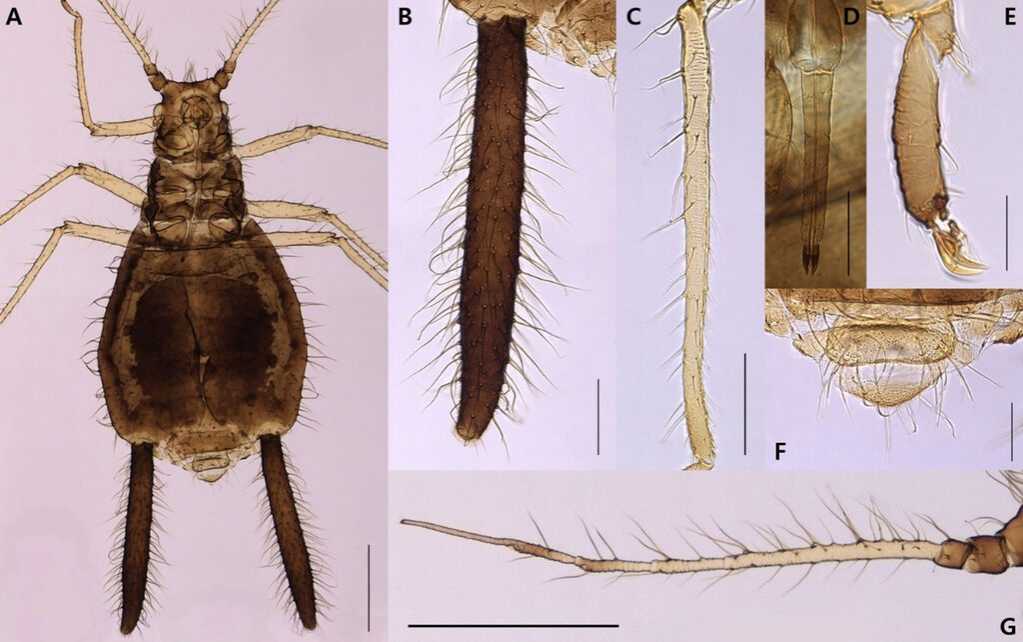
Apterous viviparous female of *Mollitrichosiphum luchuanum*. **A** Whole body; **B** SIPH; **C** HTB; **D** URS; **E** 2HT; **F** Cauda; **G** Whole Antenna. Scale bar: 500 µm (A, G); 200 µm (B, C); 100 µm (D, F); 50 µm (E).

**Apterous viviparous female** (Table 8). Color in life. Body glossy black, legs yellowish brown; distal portions of all antennae segments and tarsi dark brown in life (Fig. 14). Morphology. Body dark brown, oblong oval, 1.91-2.33 mm long (Fig. 15A). Head fused with prothorax; front 3 pairs of acute setae, 3 or 4 pairs of dorsal setae between antennae, and 3 pairs of dorsal setae between eyes. Antennae 6-segmented, Ant.Ⅰ-Ⅱ, distal end of Ⅴ-Ⅵb, and PT brown; 1.19-1.54 mm long (Fig. 15G). Long and short antennal setae are present together; longest seta on Ant.III 0.18-0.21 mm long. Ant.Ⅲ moderately transverse imbricated with 20-38 setae; Ant.Ⅳ-Ⅴ imbricated with 4-7, 4-6 setae; Ant.Ⅵ intensely imbricated with 3 or 4 setae on Ant.Ⅵb; PT 0.21-0.33 mm long, with 4 short apical setae, 1.72-2.17 times as long as Ant.Ⅵb. Distal end of rostrum passing the hind coxae; URS 0.23-0.27 mm long, with 11-14 accessory setae (Fig. 15D). Hind femur smooth, bearing slight spinules on the underside; hind tibia smooth with 41-49 transverse ridges (Fig. 15C); 2HT brown, imbricated with 7-10 setae (Fig. 15E). Abdomen spinulose ventrally; dorsal setae long and sharp, mainly pointed; ventral setae thin and fine-shaped. SIPH distinctly dark brown and long-shaped, bearing many long setae; spinulose on the both ends (Fig. 15B); 0.47-0.57 times as long as BL, 6.14-11.90 times as long as basal width of SIPH. Cauda nearly triangular with rounded apex, spinules dense with 7-9 setae (Fig. 15F); 0.40-0.45 times as long as basal width of cauda.

#### Distribution

South Korea, Japan, China (Zhang and Qiao 2010, Kang and Lee 2025a).

#### Host plants

*Meliosma myriantha*, *Meliosma rigida*, *Prunus persica*, and *Quercus* spp. (Kang and Lee 2025a, Raychaudhuri 1956, Zhang et al. 2012).

## Identification Keys

### Identification key to species of the subfamily Greenideinae in South Korea (Apterous viviparous females)

**Table:** 

1	Body pale yellow, bearing many elongated branched projections. SIPH elongated, with short setae near the tip	*Cervaphis quercus*
–	Body brown, bearing long and short setae without elongated branched projections. SIPH plump, with long setae	2
2	5-segmented antennae. SIPH not reticulated	3
–	6-segmented antennae. SIPH bearing reticulated only at the base	4
3	Body semi-glossy dark brown in life	*Eutrichosiphum pasaniae*
–	Body yellow green in life	*Eutrichosiphum sinense*
4	Abdomen smooth spinulose ventrally	*Greenidea nipponica*
–	Abdomen intensely spinulous ventrally	5
5	Hind tibia with many transverse ridges	*Mollitrichosiphum luchuanum*
–	Hind tibia without transverse ridges	6
6	SIPH 0.2-0.3 times as long as BL	*Greenidea kuwanai*
–	SIPH 0.3-0.4 times as long as BL	7
7	Dorsal setae mainly pointed. Body 1.51-1.72 times as long as body width. On *Quercus*	*Greenidea nigra*
–	Dorsal setae mainly bifurcated. Body 1.65-1.89times as long as body width. On *Castanopsis*	*Greenidea prunicola*

## Supplementary Material

XML Treatment for Cervaphis quercus

XML Treatment for Eutrichosiphum pasaniae

XML Treatment for Eutrichosiphum sinense

XML Treatment for Greenidea kuwanai

XML Treatment for Greenidea nipponica

XML Treatment for Greenidea nigra

XML Treatment for Greenidea prunicola

XML Treatment for Mollitrichosiphum luchuanum

## Figures and Tables

**Table 1.** The biometric data of apterous viviparous females *Cervaphis quercus*.

Body parts		*Cervaphis quercus* (n=14)	
		Mean	Range
Length (mm)	Body	1.65	1.49–1.76
	Body width	0.87	0.79–0.98
	Head cephalic bp	0.36	0.30–0.41
	Whole Antennae	0.60	0.56–0.65
	Ant.Ⅰ	0.05	0.04–0.06
	Ant.Ⅱ	0.05	0.05–0.06
	Ant.Ⅲ	0.27	0.24–0.30
	Ant.Ⅳb	0.09	0.08–0.10
	PT	0.14	0.12–0.15
	URS	0.20	0.18–0.22
	HFM	0.34	0.27–0.37
	HTB	0.48	0.36–0.54
	2HT	0.09	0.09–0.10
	SIPH	0.75	0.66–0.82
	SIPH BW	0.09	0.08–0.10
	SIPH DW	0.04	0.03–0.04
	Cauda	0.08	0.07–0.15
	Cauda BW	0.15	0.08–0.17
	BDAnt.Ⅲ	0.02	0.02–0.02
	Setae on Ant.Ⅲ	0.03	0.03–0.04
No. of setae on	Ant.Ⅰ	4	3–4
	Ant.Ⅱ	3	3–3
	Ant.Ⅲ	4	3–4
	URS	5	3–7
	Cauda	6	6–6
	SIPH	13	11–16
Ratio (times)	Whole Ant/BL	0.36	0.34–0.41
	PT/Ant.Ⅳb	1.46	1.18–1.60
	PT/Ant.Ⅲ	0.50	0.44–0.55
	URS/2HT	2.15	1.84–2.42
	URS/Ant.Ⅳb	2.17	1.87–2.60
	HFM/Ant.Ⅲ	1.25	0.99–1.39
	SIPH/BL	0.45	0.41–0.50
	SIPH/Ant.Ⅲ	2.72	2.54–2.88
	SIPH/SIPH BW	8.64	7.53–10.20
	SIPH/SIPH DW	19.63	17.34–21.20
	Cauda/Cauda BW	0.49	0.40–0.56
	Setae on Ant.Ⅲ/ BDAnt.Ⅲ	1.49	1.15–1.88

**Table 2.** The biometric data of apterous viviparous females *Eutrichosiphum pasaniae*.

Body parts		*Eutrichosiphum pasaniae* (n=14)	
		Mean	Range
Length (mm)	Body	1.55	1.45–1.64
	Body width	0.87	0.76–1.00
	Head width	0.37	0.33–0.39
	Whole Antennae	0.83	0.69–1.00
	Ant.Ⅰ	0.07	0.06–0.08
	Ant.Ⅱ	0.05	0.04–0.06
	Ant.Ⅲ	0.33	0.26–0.40
	Ant.Ⅳ	0.12	0.10–0.14
	Ant.Ⅴb	0.11	0.09–0.13
	PT	0.18	0.14–0.23
	URS	0.17	0.14–0.19
	HFM	0.36	0.31–0.41
	HTB	0.47	0.37–0.53
	2HT	0.09	0.08–0.09
	SIPH	0.42	0.29–0.53
	SIPH BW	0.05	0.05–0.06
	SIPH MW	0.09	0.08–0.10
	SIPH DW	0.04	0.03–0.05
	Cauda	0.05	0.04–0.06
	Cauda BW	0.15	0.12–0.16
	BDAnt.Ⅲ	0.03	0.02–0.03
	Setae on Ant.Ⅲ	0.10	0.09–0.11
No. of setae on	Ant.Ⅰ	6	5–6
	Ant.Ⅱ	5	4–6
	Ant.Ⅲ	18	12–23
	URS	8	7–10
	Cauda	7	6–8
Ratio (times)	Whole Ant/BL	0.53	0.44–0.65
	PT/Ant.Ⅴb	1.66	1.40–1.97
	PT/Ant.Ⅲ	0.53	0.46–0.61
	URS/2HT	1.88	1.69–2.08
	URS/Ant.Ⅴb	1.61	1.28–1.93
	HFM/Ant.Ⅲ	1.11	1.01–1.28
	SIPH/BL	0.27	0.19–0.34
	SIPH/Ant.Ⅲ	1.27	1.12–1.42
	SIPH/SIPH BW	7.98	5.39–9.90
	SIPH/SIPH MW	4.85	3.50–6.34
	SIPH/SIPH DW	10.36	8.62–13.86
	Cauda/Cauda BW	0.35	0.29–0.40
	Setae on Ant.Ⅲ/ BDAnt.Ⅲ	3.63	3.25–4.22

**Table 3.** The biometric data of apterous viviparous females *Eutrichosiphum sinense*

Body parts		*Eutrichosiphum sinense* (n=5)	
		Mean	Range
Length (mm)	Body	2.40	2.22–2.51
	Body width	0.88	0.80–0.95
	Head width	0.39	0.38–0.41
	Whole Antennae	1.29	1.21–1.40
	Ant.Ⅰ	0.08	0.08–0.09
	Ant.Ⅱ	0.07	0.06–0.07
	Ant.Ⅲ	0.50	0.48–0.52
	Ant.Ⅳ	0.23	0.22–0.25
	Ant.Ⅴb	0.17	0.16–0.19
	PT	0.23	0.21–0.25
	URS	0.18	0.17–0.19
	HFM	0.51	0.47–0.55
	HTB	0.72	0.67–0.79
	2HT	0.10	0.09–0.10
	SIPH	1.19	1.10–1.34
	SIPH BW	0.07	0.07–0.08
	SIPH MW	0.11	0.10–0.12
	SIPH DW	0.05	0.04–0.05
	Cauda	0.10	0.09–0.10
	Cauda BW	0.21	0.19–0.22
	BDAnt.Ⅲ	0.03	0.03–0.04
	Setae on Ant.Ⅲ	0.10	0.09–0.11
No. of setae on	Ant.Ⅰ	5	5–6
	Ant.Ⅱ	4	4–5
	Ant.Ⅲ	17	15–19
	URS	9	8–12
	Cauda	9	7–10
Ratio (times)	Whole Ant/BL	0.54	0.52–0.56
	PT/Ant.Ⅴb	1.36	1.25–1.55
	PT/Ant.Ⅲ	0.46	0.43–0.50
	URS/2HT	1.80	1.65–1.99
	URS/Ant.Ⅴb	1.04	0.92–1.14
	HFM/Ant.Ⅲ	1.01	0.97–1.08
	SIPH/BL	0.50	0.45–0.53
	SIPH/Ant.Ⅲ	2.37	2.14–2.64
	SIPH/SIPH BW	16.23	15.10–19.08
	SIPH/SIPH MW	10.53	9.47–13.31
	SIPH/SIPH DW	25.41	23.38–28.55
	Cauda/Cauda BW	0.46	0.42–0.51
	Setae on Ant.Ⅲ/ BDAnt.Ⅲ	2.97	2.73–3.23

**Table 4.** The biometric data of apterous viviparous females *Greenidea kuwanai*.

Body parts		*Greenidea kuwanai* (n=11)	
		Mean	Range
Length (mm)	Body	2.53	2.10–2.75
	Body width	1.64	1.35–1.90
	Head width	0.60	0.53–0.65
	Whole Antennae	1.58	1.29–1.81
	Ant.Ⅰ	0.11	0.09–0.12
	Ant.Ⅱ	0.07	0.06–0.09
	Ant.Ⅲ	0.58	0.42–0.66
	Ant.Ⅳ	0.18	0.14–0.22
	Ant.Ⅴ	0.19	0.14–0.23
	Ant.Ⅵb	0.15	0.13–0.17
	PT	0.32	0.27–0.36
	URS	0.26	0.23–0.29
	HFM	0.69	0.49–0.80
	HTB	0.98	0.75–1.17
	2HT	0.13	0.12–0.15
	SIPH	0.63	0.45–0.78
	SIPH BW	0.09	0.06–0.11
	SIPH MW	0.14	0.09–0.17
	SIPH DW	0.07	0.05–0.09
	Cauda	0.09	0.07–0.11
	Cauda BW	0.26	0.22–0.29
	BDAnt.Ⅲ	0.04	0.03–0.05
	Setae on Ant.Ⅲ	0.14	0.12–0.16
No. of setae on	Ant.Ⅰ	11	7–15
	Ant.Ⅱ	8	6–12
	Ant.Ⅲ	39	31–50
	URS	17	13–31
	Cauda	8	6–9
Ratio (times)	Whole Ant/BL	0.62	0.55–0.68
	PT/Ant.Ⅵb	2.14	1.79–2.29
	PT/Ant.Ⅲ	0.56	0.47–0.64
	URS/2HT	1.91	1.68–2.41
	URS/Ant.Ⅵb	1.75	1.45–2.29
	HFM/Ant.Ⅲ	1.20	1.04–1.25
	SIPH/BL	0.25	0.19–0.29
	SIPH/Ant.Ⅲ	1.10	0.96–1.22
	SIPH/SIPH BW	7.38	5.87–8.36
	SIPH/SIPH MW	4.46	3.35–5.69
	SIPH/SIPH DW	8.88	6.64–10.98
	Cauda/Cauda BW	0.36	0.28–0.42
	Setae on Ant.Ⅲ/ BDAnt.Ⅲ	3.48	2.97–3.96

**Table 5.** The biometric data of apterous viviparous females *Greenidea nipponica*.

Body parts		*Greenidea nipponica* (n=1)	
		Mean	
Length (mm)	Body		2.82
	Body width		1.76
	Head width		0.64
	Whole Antennae		1.71
	Ant.Ⅰ		0.11
	Ant.Ⅱ		0.07
	Ant.Ⅲ		0.61
	Ant.Ⅳ		0.21
	Ant.Ⅴ		0.22
	Ant.Ⅵb		0.16
	PT		0.36
	URS		0.27
	HFM		0.72
	HTB		1.05
	2HT		0.14
	SIPH		0.78
	SIPH BW		0.09
	SIPH MW		0.17
	SIPH DW		0.07
	Cauda		0.10
	Cauda BW		0.25
	BDAnt.Ⅲ		0.05
	Setae on Ant.Ⅲ		0.16
No. of setae on	Ant.Ⅰ		11
	Ant.Ⅱ		6
	Ant.Ⅲ		39
	URS		18
	Cauda		7
Ratio (times)	Whole Ant/BL		0.61
	PT/Ant.Ⅵb		2.28
	PT/Ant.Ⅲ		0.59
	URS/2HT		1.92
	URS/Ant.Ⅵb		1.72
	HFM/Ant.Ⅲ		1.17
	SIPH/BL		0.28
	SIPH/Ant.Ⅲ		1.28
	SIPH/SIPH BW		8.68
	SIPH/SIPH MW		4.62
	SIPH/SIPH DW		10.43
	Cauda/Cauda BW		0.38
	Setae on Ant.Ⅲ/ BDAnt.Ⅲ		3.55

**Table 6.** The biometric data of apterous viviparous females *Greenidea nigra*.

Body parts		*Greenidea nigra* (n=12)	
		Mean	Range
Length (mm)	Body	2.64	2.11–2.89
	Body width	1.65	1.29–1.92
	Head width	0.62	0.55–0.67
	Whole Antennae	1.76	1.52–1.96
	Ant.Ⅰ	0.12	0.10–0.14
	Ant.Ⅱ	0.08	0.06–0.10
	Ant.Ⅲ	0.65	0.55–0.72
	Ant.Ⅳ	0.21	0.17–0.24
	Ant.Ⅴ	0.22	0.19–0.24
	Ant.Ⅵb	0.16	0.12–0.18
	PT	0.36	0.28–0.42
	URS	0.25	0.23–0.26
	HFM	0.79	0.64–0.90
	HTB	1.12	0.91–1.33
	2HT	0.14	0.12–0.15
	SIPH	0.89	0.75–1.03
	SIPH BW	0.11	0.09–0.12
	SIPH MW	0.16	0.12–0.18
	SIPH DW	0.08	0.07–0.09
	Cauda	0.10	0.08–0.12
	Cauda BW	0.26	0.23–0.28
	BDAnt.Ⅲ	0.05	0.04–0.06
	Setae on Ant.Ⅲ	0.19	0.17–0.21
No. of setae on	Ant.Ⅰ	9	7–12
	Ant.Ⅱ	8	7–9
	Ant.Ⅲ	41	35–48
	URS	16	14–20
	Cauda	6	6–8
Ratio (times)	Whole Ant/BL	0.67	0.61–0.72
	PT/Ant.Ⅵb	2.23	1.77–2.69
	PT/Ant.Ⅲ	0.55	0.51–0.61
	URS/2HT	1.70	1.58–1.86
	URS/Ant.Ⅵb	1.54	1.39–1.89
	HFM/Ant.Ⅲ	1.21	1.13–1.31
	SIPH/BL	0.34	0.28–0.39
	SIPH/Ant.Ⅲ	1.37	1.24–1.49
	SIPH/SIPH BW	8.35	7.54–9.56
	SIPH/SIPH MW	5.73	5.04–6.75
	SIPH/SIPH DW	11.20	10.09–12.77
	Cauda/Cauda BW	0.39	0.35–0.44
	Setae on Ant.Ⅲ/ BDAnt.Ⅲ	3.90	3.27–4.52

**Table 7.** The biometric data of apterous viviparous females *Greenidea prunicola*.

Body parts		*Greenidea prunicola* (n=10)	
		Mean	Range
Length (mm)	Body	2.23	1.70–2.60
	Body width	1.25	0.94–1.57
	Head width	0.53	0.43–0.60
	Whole Antennae	1.92	1.47–2.25
	Ant.Ⅰ	0.11	0.09–0.12
	Ant.Ⅱ	0.07	0.06–0.08
	Ant.Ⅲ	0.48	0.35–0.57
	Ant.Ⅳ	0.27	0.21–0.31
	Ant.Ⅴ	0.29	0.22–0.36
	Ant.Ⅵb	0.22	0.17–0.26
	PT	0.49	0.37–0.56
	URS	0.20	0.18–0.23
	HFM	0.65	0.47–0.77
	HTB	0.86	0.59–1.02
	2HT	0.12	0.10–0.13
	SIPH	0.76	0.57–0.93
	SIPH BW	0.07	0.06–0.08
	SIPH MW	0.12	0.11–0.13
	SIPH DW	0.06	0.05–0.06
	Cauda	0.09	0.07–0.10
	Cauda BW	0.21	0.16–0.23
	BDAnt.Ⅲ	0.04	0.03–0.04
	Setae on Ant.Ⅲ	0.11	0.10–0.13
No. of setae on	Ant.Ⅰ	8	7–10
	Ant.Ⅱ	6	4–7
	Ant.Ⅲ	17	15–21
	URS	14	12–16
	Cauda	8	6–8
Ratio (times)	Whole Ant/BL	0.86	0.78–0.90
	PT/Ant.Ⅵb	2.27	2.10–2.51
	PT/Ant.Ⅲ	1.02	0.91–1.15
	URS/2HT	1.66	1.53–1.85
	URS/Ant.Ⅵb	0.92	0.81–1.05
	HFM/Ant.Ⅲ	1.34	1.30–1.41
	SIPH/BL	0.34	0.32–0.37
	SIPH/Ant.Ⅲ	1.58	1.42–1.65
	SIPH/SIPH BW	10.74	9.50–12.03
	SIPH/SIPH MW	6.36	5.16–7.30
	SIPH/SIPH DW	13.54	11.27–15.60
	Cauda/Cauda BW	0.42	0.34–0.48
	Setae on Ant.Ⅲ/ BDAnt.Ⅲ	3.15	2.69–3.57

**Table 8.** The biometric data of *Mollitrichosiphum luchuanum*.

Body parts		*Mollitrichosiphum luchuanum* (n=12)	
		Mean	Range
Length (mm)	Body	2.21	1.91–2.33
	Body width	1.10	0.92–1.21
	Head width	0.44	0.40–0.46
	Whole Antennae	1.44	1.19–1.54
	Ant.Ⅰ	0.08	0.07–0.09
	Ant.Ⅱ	0.07	0.06–0.08
	Ant.Ⅲ	0.51	0.40–0.56
	Ant.Ⅳ	0.17	0.15–0.18
	Ant.Ⅴ	0.19	0.16–0.21
	Ant.Ⅵb	0.15	0.12–0.16
	PT	0.29	0.21–0.33
	HFM	0.54	0.44–0.59
	HTB	0.89	0.75–0.94
	2HT	0.13	0.12–0.14
	URS	0.25	0.23–0.27
	SIPH	1.18	0.97–1.32
	SIPH BW	0.12	0.10–0.20
	SIPH MW	0.15	0.13–0.17
	SIPH DW	0.08	0.08–0.10
	Cauda	0.07	0.07–0.08
	Cauda BW	0.18	0.16–0.19
	BDAnt.Ⅲ	0.04	0.04–0.05
	Setae on Ant.Ⅲ	0.20	0.18–0.21
No. of setae on	Ant.Ⅰ	6	5–7
	Ant.Ⅱ	4	3–5
	Ant.Ⅲ	33	20–38
	URS	13	11–14
	Cauda	8	7–9
Ratio (times)	Whole Ant/BL	0.65	0.58–0.70
	PT/Ant.Ⅵb	1.95	1.72–2.17
	PT/Ant.Ⅲ	0.56	0.52–0.64
	URS/2HT	1.95	1.78–2.08
	URS/Ant.Ⅵb	1.72	1.61–1.89
	HFM/Ant.Ⅲ	1.06	0.97–1.24
	SIPH/BL	0.53	0.47–0.57
	SIPH/Ant.Ⅲ	2.30	2.18–2.44
	SIPH/SIPH BW	10.01	6.14–11.90
	SIPH/SIPH MW	7.69	7.24–8.97
	SIPH/SIPH DW	14.43	12.05–17.12
	Cauda/ Cauda BW	0.42	0.40–0.45
	Setae on Ant.Ⅲ/ BDAnt.Ⅲ	4.75	4.12–5.47
